# Glioblastoma patients in Slovenia from 1997 to 2008

**DOI:** 10.2478/raon-2014-0002

**Published:** 2014-01-22

**Authors:** Uros Smrdel, Viljem Kovac, Mara Popovic, Matjaz Zwitter

**Affiliations:** 1 Department of Radiotherapy, Institute of Oncology Ljubljana, Ljubljana, Slovenia; 2 Institute of Pathology, Faculty of Medicine, University of Ljubljana, Slovenia

**Keywords:** glioblastoma, treatment, survival, surgery, radiotherapy, temozolomide

## Abstract

**Background:**

Glioblastoma is the most common primary brain tumour. It has a poor prognosis despite some advances in treatment that have been achieved over the last ten years. In Slovenia, 50 to 60 glioblastoma patients are diagnosed each year. In order to establish whether the current treatment options have any influence on the survival of the Slovenian glioblastoma patients, their data in the period from the beginning of the year 1997 to the end of the year 2008 have been analysed.

**Patients and methods:**

All patients treated at the Institute of Oncology Ljubljana from 1997 to 2008 were included in the retrospective study. Demographics, treatment details, and survival time after the diagnosis were collected and statistically analysed for the group as a whole and for subgroups.

**Results:**

From 1997 to 2008, 527 adult patients were diagnosed with glioblastoma and referred to the Institute of Oncology for further treatment. Their median age was 59 years (from 20 to 85) and all but one had the diagnosis confirmed by a pathologist. Gross total resection was reported by surgeons in 261 (49.5%) patients; good functional status (WHO 0 or 1) after surgery was observed in 336 (63.7%) patients, radiotherapy was performed in 422 (80.1%) patients, in 317 (75.1%) of them with radical intent, and 198 (62.5 %) of those received some form of systemic treatment (usually temozolomide). The median survival of all patients amounted to 9.7 months. There was no difference in median survival of all patients or of all treated patients before or after the chemo-radiotherapy era. However, the overall survival of patients treated with radical intent was significantly better (11.4 months; p < 0.05). A better survival was also noticed in radically treated patients who received additional temozolomide therapy (11.4 *vs*. 13.1 months; p = 0.014). The longer survival was associated with a younger age and a good performance status as well as with a more extensive tumour resection. In patients treated with radical intent, having a good performance status, and receiving radiotherapy and additional temozolomide therapy, the survival was significantly longer, based on multivariate analysis.

**Conclusions:**

We observed a gradual increase in the survival of glioblastoma patients who were treated with radical intent over the last ten years. Good functional surgery, advances in radiotherapy and addition of temozolomide all contributed to this increase. Though the increased survival seems to be more pronounced in certain subgroups, we have still not been able to exactly define them. Further research, especially in tumour biology and genetics is needed.

## Introduction

Glioblastoma (GBM) is a WHO grade IV tumour arising from the astrocytes and represents the most common type of primary central nervous system (CNS) malignancies providing for well over one half of all gliomas.^1^,^2^ GBM is characterised by a rapid growth and short time to progression in most treated patients. Its peak incidence is in the sixth and seventh decades of life with much lower incidence in younger age groups where low-grade gliomas predominate. It can also appear in the childhood, although the most frequent brain tumour in this period of life is meduloblastoma.^3^–^8^

Though the incidence of malignant gliomas is increasing among the elderly^8^,^9^, the age-adjusted incidence of GBM in Slovenia remained comparatively low in the range of 2.3 to 3.0 per 100000 per year. This brought about around 50 new cases each year.^10^

GBM is one of the tumours least likely to be cured and causes quite severe physical as well as cognitive and psychological disabilities. Thus, it has a significant impact on the lives of affected patients, their caregivers and relatives, which is not to be underestimated.

By the introduction of a combined modality treatment, a statistically significant increase of survival in GBM patients was observed. This can in part be contributed to the addition of systemic therapy, but also to the improved quality of radiation therapy resulting in an increase of the total dose given.^9^,^11^,^12^ There was a tendency to treat more patients who had only been deemed suitable for supportive care in the past.^13^,^14^ Therefore palliative irradiation was applied to the tumour bed with a slightly higher dose than it was the case with meta-static brain tumours^15^–^17^, and a less toxic systemic treatment was prescribed sequentially.^18^,^19^

Classically, younger GBM patients tended to fare better than older ones and, in some cases, patients over the age of seventy were treated palliatively and did not receive the same amount of treatment as younger ones. Likewise, although the performance status was one of the more important prognostic factors, it tended not to be taken into account when younger and older patients were treated. Therefore often even quite fit elderly patients received suboptimal treatment while severely ill younger patients were treated with aggressive regimens.^20^–^22^

The cause of gliomas is unknown though various theories has suggested a wide range of possible causes from nonionizing radiation to viral aetiology, none has as yet been proven, ionizing radiation aside. The sequence of mutations leading to the tumour is reasonably well known, with the key events in tumour genesis well documented.^23^–^30^

The diagnosis is usually established after a short period of complaints, headache being most frequent but not obligatory. In fact, only around 60% of patients report headache, less frequent are convulsions and focal neurological disturbances. On imaging, there is usually a contrast-enhancing tumour with varied signal surrounded by oedema. Neurosurgery should be applied for therapeutic and diagnostic purposes. A microscopic examination of the tumour specimen acquired by neurosurgery is necessary for a definite diagnosis.^31^,^32^ Often, surgery is performed without the intention of resection or even reduction, the only goal being the biopsy and microscopic diagnosis, though some recent studies suggest that the extent of the tumour resection is one of the important factors influencing the survival of the patients. Therefore, a maximal safe tumour resection is mostly recommended.^33^

After surgery, the patients were typically treated with radiotherapy with or without systemic therapy.^9^,^34^–^38^ Radiotherapy is usually performed at a dose level between 55 and 60 Gy applied at the tumour site with an additional 2 to 3 cm margin at preoperative MRI, 5 times weekly. Since 2004, concomitant radio-chemotherapy has been applied in the treatment of GBM patients. Usually, patients receive the same dose of radiotherapy, but with the addition of temozolomide in doses of 75 mg/m^2^ daily during radiotherapy, followed by adjuvant temozolomide. These procedures are likely to produce an overall survival of 1 to 1.5 years with a 2-year survival of around 25% of the patients.^11^,^39^

In this paper, we are trying to review and evaluate the Slovenian GBM treatment results over 14 years along with the current treatment options while addressing some questions arising.

## Patients and methods

### Patients

In the retrospective study we analysed the treatments of glioblastoma patients at the Institute of Oncology in Ljubljana and their survival in the period from 1997 to 2008. There were included all glioblastoma patients treated with any other treatment modality but surgery alone and, for the period after 2000, there were included all patients fit enough for any therapy to be considered.

In Slovenia, most hospitals have neurology departments and internal medicine departments performing initial diagnosis of CNS neoplasms. Patients with unknown extra cranial primary were referred either to one of the two neurosurgical departments in Ljubljana and Maribor for surgical treatment or, in accordance with the imaging, if a brain metastasis was suspected and there was no immediate need for debulking surgery, to an appropriate diagnostic department.

After surgery, the microscopic examination of the tumour specimens was performed at one of the two pathology departments in Ljubljana and Maribor. Subsequently, all patients were discussed at multidisciplinary team meetings and the treatment strategy was outlined.

The vast majority of patients, with the exception of those deemed to be unfit to receive any further treatment, were referred to the Institute of Oncology for additional treatment. All patients in Slovenia were post-operatively treated in only one institution.

In the recent years patients deemed suitable for radiotherapy were treated either with 60 Gy in 30 fractions or with 59.4 Gy in 33 fractions over 6 to 7 weeks. Those intended for palliative irradiation received 35 Gy in 10 fractions or 30 Gy in 6 fractions over two weeks. All patients received radiotherapy at the linear accelerator, yet until 2005 some patients were treated at the old cobalt unit. Since 2004 all treatments were planned at 3D conformal (XiO and Eclipse). Prior to that, radical patients had been treated with plan done in one axial plane using Multidata TPS, without taking tissue inhomogeneity into account. All others had a simple depth dose calculation using in-house software. Since November 2004, the patients with good performance status were offered a possibility of concomitant chemo-radiotherapy with temozolomide followed by adjuvant chemotherapy over 6 or more cycles, depending on tumour response and toxicity.^40^–^48^

At progression, patients were repeatedly discussed at a multidisciplinary team meeting. If surgical treatment was possible, the patients were referred back to the neurosurgeon, or second-line systemic therapy was offered to the patients with good performance status. For patients relapsing after a period of more than a year, re-irradiation was considered, while others received supportive and symptomatic treatments.

### Methods

The data were obtained from the hospital registry of the Institute of Oncology, from the Cancer Registry of Slovenia and from the treatment charts.

The data collected were the age of the patients at the diagnosis, the sex, the extent of neurosurgery, the performance status after surgery, the radiotherapy parameters (total dose, dose per fraction, number of fractions and treatment planning) and systemic therapy.

We calculated the overall survival from the day of diagnosis to the death of all patients or when censored. The Kaplan-Meier method was used for the estimation of overall survival and the log-rank test was used to compare survival distributions between samples. A p-value lower than 0.05 was considered as statistically significant. Statistical analysis was performed using SPSS statistical package (Release 19.0, IBM SPSS).

The investigators strictly followed recommendations of the Helsinki Declaration and of the Council of Europe Convention on Human Rights and Biomedicine.

## Results

### Patients

In the period from the beginning of 1997 to the end of 2008, Cancer Registry of Slovenia registered 1145 patients with primary CNS tumours, 527 (46%) of them were GBM patients treated at the Institute of Oncology Ljubljana.

The patients’ age ranged from 20 to 85 years, with median age of 59 years with a standard deviation (SD) of 11.8 years. The median age of patients slowly increased from 54 in 1997 to 63 in 2008 (Table 1).

During the period examined, the number of the glioma patients treated at the Institute of Oncology slightly increased. In 1997, 27 patients were treated and then the number slowly grew. It settled at around 55 patients per year.

### Diagnosis and surgical treatment

All but one patient had the diagnosis confirmed by means of microscopic examination of surgically removed tumour samples. In one half of the patients (261 out of 527; 49.5%) the surgeons reported a gross total resection, in 191 (36.2%) patients the tumour was reduced and in 74 (14.0%) patients only diagnostic biopsy was performed (Table 2). The number of biopsies only was constantly low throughout the observed period.

The surgical results regarding the patients’ performance status were good, with almost two thirds of the patients (336; 63.7%) having the WHO performance status of 0 and 1.

### Radiotherapy

Most patients (422 out of 527; 80.1%) received some kind of radiotherapy, with either palliative or radical intent (317). Unfortunately, it was not possible for all 317 patients treated with radical intent to complete the radiotherapy. Some of them deteriorated during treatment. An equal number of patients received either palliative treatment or best supportive care.

In the patients receiving radiotherapy, the median tumour dose (TD) was 50 Gy, with SD of 10 Gy (range 2–67.5 Gy). In the patients irradiated with radical intent, the median TD was 56 Gy with SD of 8.4 Gy (range 2–65.7 Gy). In palliative patients, the median TD was 37.5 Gy with SD of 6.8 Gy (range 6–46 Gy), the most common fractionation being 35 Gy in 10 fractions and 45 Gy in 15 fractions. In patients receiving radical intent treatment, there was a trend for a gradual dose increase from 42 Gy through 50 Gy and 56 Gy to the now usual dose of 60 Gy. In total, the mean dose increased from 44.2 Gy in 1997 to 57.3 Gy in 2008.

### Systemic therapy

From 1997 to the publication of EORTC study results in 2004, 301 glioma patients were seen at the Institute of Oncology Ljubljana. Two of them were enrolled in the trial and received concomitant chemo-radiotherapy, 46 out of 301 (15.3%) received adjuvant chemotherapy with temozolomide and 10 out of 301 (3.3%) received other forms of chemotherapy (carmustine (BCNU) or procarbazine, lomustine, vincristine (PCV). Two hundred forty-three out of 301 (80.7%) patients received no systemic treatment. From the publication of the EORTC study results in 2004 to the year 2008, 266 glioblastoma patients were referred to the Institute of Oncology, one half of which (122; 54%) received concomitant chemo-radiotherapy followed by adjuvant chemotherapy with temozolomide. Over the last period, 16 (7.1%) patients received adjuvant temozolomide only, 1 (%) patient received BCNU only, and 87 (38.5%) patients received no systemic treatment (Table 2).

### Survival

**FIGURE 1.**
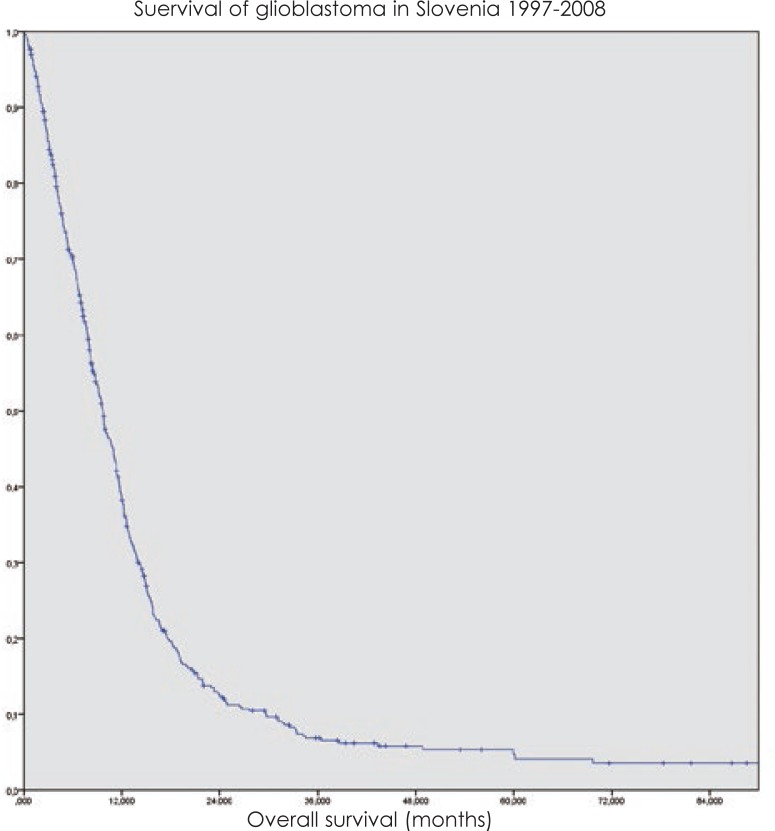
Overall survival of glioblastoma patients in Slovenia treated at Institute of Oncology Ljubljana from 1997 to 2008.

The overall median survival of the whole group was 9.7 months with standard deviation (SD) of 0.53 months (Figure 1).

There was no difference in median survival either of all patients or of all treated patients before and after chemo-radiotherapy era. However, the overall survival of the patients treated with radical intent radiotherapy was significantly longer (p < 0.05). Before the introduction of chemo-radiotherapy, the overall median survival of these patients was 11.4 months and afterwards it amounted to 13.1 months (p = 0.014).

**FIGURE 2.**
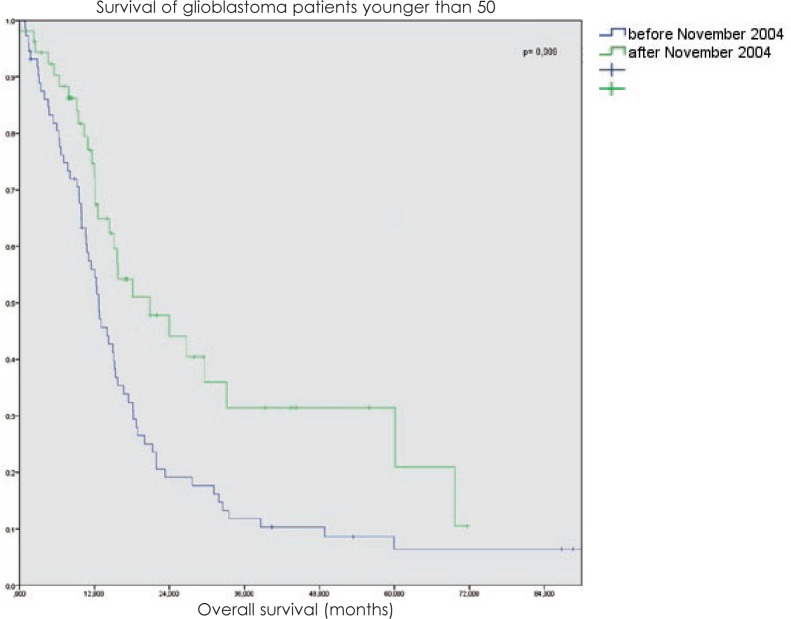
Overall survival of glioblastoma patients under 50-year radically treated at Institute of Oncology Ljubljana from 1997 to 2008.

The benefit of radical intent radiotherapy followed by chemotherapy was even more evident in the group of patients younger than 50 years with the median survival rising from 14.9 months to 24 months (p = 0.009) and with 26% patients surviving even more than 48 months (Figure 2).

Likewise, there was some survival benefit associated with more extensive surgery. The median survival of the patients with gross total resection of the tumour was 14.4 months, with partial surgical reduction 11.4 months and with biopsy only 8.4 months (p = 0.088).

The patients with good performance status after surgery had a median survival of 13.8 months, while those with a WHO performance status of 2 or 3 had a median survival of 9.8 months (p < 0.001).

In multivariate analysis, an age below 50 years, a gross total resection, a good performance status and chemo-radiotherapy all were associated with longer survival (p < 0.05).

**FIGURE 3.**
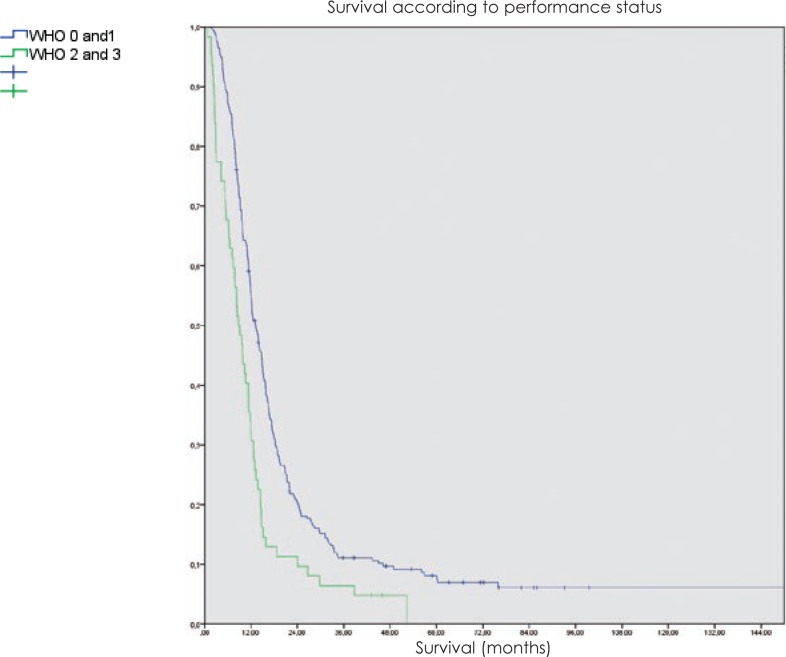
Overall survival of glioblastoma patients treated with radiotherapy at Institute of Oncology Ljubljana from 1997 to 2008 according to performance status.

In patients treated with radiotherapy, there was no difference in survival connected to the extent of surgery in the group of patients younger than 50 years, but in the older patient group, there was a marked improvement of survival of those with gross total resection of the tumour (p < 0.05). In both age groups, the performance status after surgery was an important factor, even more so in the younger patients (Figure 3).

In the younger patients the effect of radiation techniques was more important. They greatly benefited from the introduction of more complex treatments and from the increase of the total dose with a median survival of 20.8 vs. 12.7 months (p = 0.02).

**FIGURE 4.**
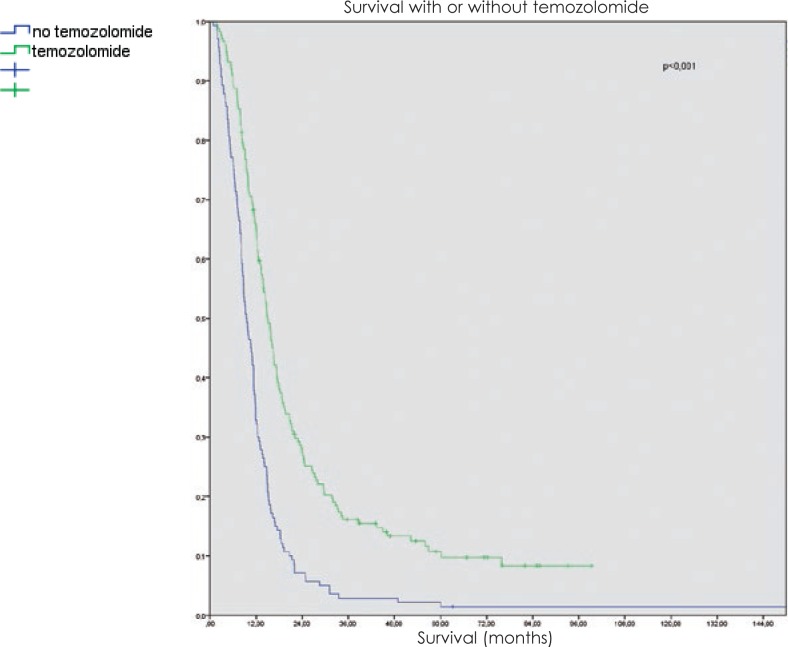
Overall survival of glioblastoma patients according the treatment with temozolomide at Institute of Oncology Ljubljana from 1997 to 2008.

The survival was improved with the addition of temozolomide. While the median survival was only around 9 months in radically treated patients without chemotherapy, even the addition of temozolomide in adjuvant setting improved the median survival to 14 months, and the concomitant treatment followed by adjuvant temozolomide increased it to 16 months. In the age group under 50 years, the impact of concomitant treatment was even greater (16.7 months with adjuvant treatment *vs.* 20.8 months after concomitant treatment followed by adjuvant temozolomide). In the age group over 50 years, there was virtually no difference in survival regardless of temozolomide schedule (Figure 4).

**FIGURE 5.**
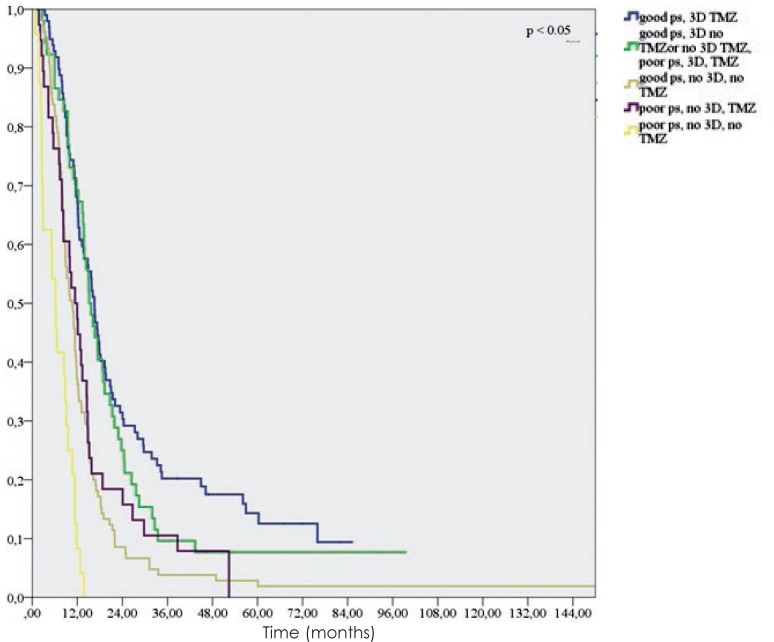
Overall survival of glioblastoma patients treated with radiotherapy at Institute of Oncology Ljubljana from 1997 to 2008 according to performance status, radiotherapy planning and addition of temozolomide.

In the group of patients treated with radiotherapy with a “radical” dose (317 patients), the extent of surgery played no significant role (p = 0.179). On the other hand, we found that in this group good performance status (WHO 0 and 1 *vs.* 2 and 3) after surgery (p < 0.005), radiotherapy planning (depth dose, 2D *vs.* 3D conformal) (p = 0.015) and the addition of temozolomide (p < 0.005) were statistically significant (Figure 5).

## Discussion

In our institution, the median survival of GBM patients since 1997 has risen similarly as elsewhere in the world. While the diagnosis of GBM remains one of the most unfavourable ones, there has been an increase of the overall survival and also of the time to progression observed in the patients treated for GBM in the last ten years.^49^ Though the largest increase of survival was achieved by the inclusion of temozolomide in the initial treatment of GBM, there seems to be a subgroup of patients who benefited more than others.

In our analysis, we could confirm a gradual and modest increase in the overall survival of glioblastoma patients. Any potential changes in the time to progression were harder to detect, especially since our earliest patients were usually discharged from follow up after initial treatment or visited the clinic only once at the most after the completion of radiotherapy treatment and no imaging was performed. Some patients originated from other republics of the former Yugoslavia and for them even the overall survival was somewhat doubtful, so the overall survival in particular cases might have been more than calculated.

We observed a positive impact on survival after concomitant chemo-radiotherapy was introduced in 2004.^34^ The survival increased most in the subgroup of younger patients with a maximal safe removal of the tumour, with a good post-operative performance status, receiving a dose of 56 to 60 Gy with 3D conformal radiotherapy as well as receiving concomitant and adjuvant temozolomide. Unfortunately, they were not cured. The majority of them progressed in due course. However, they were mostly exposed to some sort of treatment after progression. The salvage therapy seemed to work for the majority of those progressing after a prolonged progression-free survival.^50^

In the older age group, there was also an impact of combined modality treatment on survival. It is interesting that in our group the impact of concomitant treatment in this group was less pronounced. The patients receiving only adjuvant treatment seemed to fare no worse than those in the concomitant treatment group. This could probably be explained by the lesser toxicity of the former during initial treatment.^51^,^52^

In the present analysis, almost 10% of the patients treated with radical intent stayed alive for more than 3 years. This was somewhat more than usually reported. On average, they were younger patients, but still one third of them were over 50. On the other hand, one quarter of those who survived less than a year were under 50. Therefore, it appears unfair to deny the best possible treatment to patients just on the basis of their age and regardless of the patients’ performance status.

A poor performance status after surgery was clearly linked with a poor prognosis, yet some patients, especially younger ones, had at least an average survival nevertheless. Thus, age seems to be more important in patients with a poor performance status than in overall GBM patients.

While it is clear from our results that there were clinical factors correlating with the longer survival of GBM patients, there is a need to find out other possible influences. The patients’ age, extent of surgery and performance status after surgery might fail to identify patients suitable for a particular kind of treatment in the primary as well as in the secondary setting. In addition to the changes in radiotherapy planning and delivery, systemic therapy schedules and potential impact of salvage therapy, some exciting new possibilities are emerging in the field of molecular biology and genetics of GBM, including the biomarkers, which could help to determine the risks of a particular patient and to tailor his treatment accordingly.^53^–^56^ Moreover, while targeted therapy is on the rise in other fields of oncology and vascular endothelial growth factor receptor (VEGFR) and integrin inhibitors are becoming available, we still do not know how to select patients for the application of these modalities and if there is a subgroup of patients who would benefit from a different initial approach than the one used today. All those questions will need to be answered in the near future.^57^,^58^

## Conclusions

GBM remains a challenging issue for the patients, their immediate caregivers and for medical personnel. Since the introduction of temozolomide in early 2000s, there have been only minor advances. For some patients this treatment clearly seems to be rather advantageous and a larger proportion of patients than before survived over two years.

In this respect, Slovenia is no exception. Even more than elsewhere, neurosurgeons are performing extensive resections. Having only one oncology centre helps to assure an equal treatment of similar patients, yet a number of questions remain unanswered. For example, is it possible to select patients responding well to the current treatment and how can an alternative be offered to those not benefiting from it? Who are those patients and what is the alternative?

## Figures and Tables

**TABLE 1.** Characteristics of glioblastoma patients in Slovenia treated at Institute of Oncology Ljubljana from 1997 to 2008

**Characteristics**	**Patients, n = 527 (%)**
Age (years)	59 (SD 11.8)
Minimum	20
Maximum	85
Gender	
Males	321 (60.9)
Females	206 (39.1)
Performance status (WHO)	
0	84 (15.9)
1	252 (47.8)
2	103 (19.5)
3	86 (16.3)
4	2 (0.4)

**TABLE 2.** Treatment characteristics of glioblastoma patients in Slovenia referred to Institute of Oncology Ljubljana from 1997 to 2008

**Treatment**	**Patients, n = 527 (%)**
Surgery	
Gross total resection	261 (49.5)
Reduction	191 (36.2)
Biopsy	74 (14.0)
None	1 (0.2)
Radiotherapy	422 (80.1)
Palliative	105 (24.9)
Radical	317 (75.1)
None	104 (19.9)
Chemotherapy	198 (37.4)
Temozolomide	187 (94.9)
Concomitant	125 (66.8)
Adjuvant	62 (33.1)
Other (BCNU, PCV)	11 (5.1)
None	330 (62.6)

BCNU = carmustine; PCV = procarbazine, lomustine, vincristine
